# Health Technology Assessment capacity development in low- and middle-income countries: Experiences from the international units of HITAP and NICE

**DOI:** 10.12688/f1000research.13180.1

**Published:** 2017-12-11

**Authors:** Sripen Tantivess, Kalipso Chalkidou, Nattha Tritasavit, Yot Teerawattananon

**Affiliations:** 1 Health Intervention and Technology Assessment Program (HITAP), Ministry of Public Health, Thailand, Muang, Nonthaburi, 11000, Thailand; 2 Center for Global Development, London, SW1Y 4TE, UK; 3 International Unit, Health Intervention and Technology Assessment Program (HITAP), Ministry of Public Health, Thailand, Muang, Nonthaburi, 11000, Thailand

**Keywords:** priority setting, health technology assessment, health policy, capacity building, low- and middle-income countries

## Abstract

Health Technology Assessment (HTA) is policy research that aims to inform priority setting and resource allocation. HTA is increasingly recognized as a useful policy tool in low- and middle-income countries (LMICs), where there is a substantial need for evidence to guide Universal Health Coverage policies, such as benefit coverage, quality improvement interventions and quality standards, all of which aim at improving the efficiency and equity of the healthcare system.

The Health Intervention and Technology Assessment Program (HITAP), Thailand, and the National Institute for Health and Care Excellence (NICE), UK, are national HTA organizations providing technical support to governments in LMICs to build up their priority setting capacity. This paper draws lessons from their capacity building programs in India, Colombia, Myanmar, the Philippines, and Vietnam. Such experiences suggest that it is not only technical capacity, for example analytical techniques for conducting economic evaluation, but also management, coordination and communication capacity that support the generation and use of HTA evidence in the respective settings. The learned lessons may help guide the development of HTA capacity in other LMICs.

## Introduction

Health technology assessment (HTA) has been widely recognized as a policy tool, which provides helpful information for allocating finite resources and ensures equitable access to needed technologies in the context of universal health coverage (UHC) ^1^. HTA determines effects and implications of a variety of technology, not only medicines, vaccines, medical devices and procedures, but also social interventions whose aim is to improve health ^2^. HTA involves research in multiple disciplines in order to assess cost effectiveness, budget impact, programmatic feasibility and social and ethical issues of health interventions ^3^. The demand for HTA capacity in low- and middle-income countries (LMICs) is likely to grow, partly due to the adoption of the World Health Organization (WHO) regional committees’ and World Health Assembly resolutions during 2012 to 2014 ^4– 6^.

HTA and its policy utilization is well established in high-income countries, such as Australia, Canada, and European countries ^7^. On the contrary, HTA capacity in most LMICs are lacking, especially in making links between evidence and policy ^8– 10^. In many settings where UHC has been adopted as a national policy, policymakers express their concerns about the financial sustainability of the healthcare services and also the demand for priority setting tools ^11^. However, local research capacity is inadequate to supply HTA evidence ^12^, and the connection between research and policy is impeded by several factors, such as the lack of awareness, understanding, knowledge and adequate will on the part of policymakers, technical officers and researchers on HTA and its role in evidence-based decisions.

During the past decade, capacity building programs for HTA have been initiated by international and regional organizations and networks. These programs usually involve short-course trainings for academics and policy analysts, convening of annual conferences, and development and distribution of HTA guides, such as methodological guidance ^13– 15^. Furthermore, regional networks mostly active in Asia (HTAsiaLink) and Latin America (RedETSA, PAHO and IADB) have helped share experiences on HTA between countries ^a^. In late 2000s, the UK’s National Institute for Health and Care Excellence (NICE) International and Thailand’s Health Intervention and Technology Assessment Program (HITAP) and their partner organizations agreed to establish partnerships to strengthen priority setting in LMICs in different regions. 

While health priority setting is indispensable, lessons drawing on the experiences of NICE and HITAP will be helpful for introduction of HTA at country level. This paper reviews the efforts of the two institutes to encourage evidence generation and utilization of research in decision making in five LMICs. It also discusses the system context, including supportive factors of and challenges in the capacity building mission for each study setting. 

## Introduction to NICE and HITAP, and their international units

### NICE and NICE International

The UK’s National Institute for Health and Clinical Excellence (NICE) was set up in 1999, an integral part of the government’s aim to introduce clinical governance, and improve the quality, equity and efficiency of a chronically underfunded National Health Service (NHS) ^16^. In addition to a commitment to using scientific evidence of comparative clinical and cost effectiveness, NICE built up its core decision making processes based on the principles of multistakeholder engagement, transparency, conflict of interest management and contestability. Over the years its remit grew to include deciding on coverage of all new drugs introduced in the NHS, setting quality standards for all core services, including safe staffing levels, and issuing guidance on public health and social care.

In the UK, the role of independent institutions such as NICE in translating evidence into policy (‘knowledge brokers’) has been highlighted before ^b^ as has the application of this model of institutional capacity well beyond health, in social policy and practice, through the country’s What Works Evidence Centers ^c^.

As NICE’s international reputation grew so did requests from overseas policy makers for support in strengthening their own systems and processes for making difficult decisions on how best to allocate their limited budgets. In response, NICE International was set up in 2008 to offer: “Advice on building capacity for assessing and interpreting evidence to inform health policy and on designing and using methods and processes to apply this capacity” for better health around the world through effective and equitable use of resources.

### HITAP and HITAP International Unit

Established in 2007, HITAP is a semi-autonomous research arm of Thailand’s Ministry of Health (MOH). Its main mission is to conduct HTA and provide evidence and recommendations to decision makers ^17^. HITAP’s research, including cost-effectiveness studies and budget impact analysis, has been formally embedded as part of coverage decisions, i.e. development of reimbursable medicines list and benefit package of the Universal Health Coverage scheme (a government-financed health scheme for 75% of population). During the past eight years, over 150 studies were conducted in this institute; most of them have been fed into national policymaking process ^18, 19^. Besides the policy analysis role, HITAP implements strategies to support HTA introduction in the country, such as capacity building for HTA researchers and users; development of guidelines, tools and a database of studies; and knowledge management strategies. In 2013, HITAP International Unit (HIU) was established to coordinate research, capacity building, research dissemination, networking and other activities at regional and international levels.

### Country partners of NICE International and HITAP

As of 2015, NICE International works in 7 countries in Europe, Latin America, Africa, and Asia, while HIU works in 7 countries in Asia. The two institutes apply similar principles of selecting countries with which they work. First, the LMIC should have a goal to move toward UHC and a policy demand for HTA capacity building; second, government or not-for-profit organizations assigned as a focal point committed to absorb capacity building; third, local partners agree to work on policy-relevant case studies or pilot projects in a participatory, and transparent manner. Furthermore, NICE International and HITAP give a higher priority to countries that will enable their staff to learn more on technical and policy issues.

## Conceptual framework for HTA capacity development in LMICs

**Figure 1.**
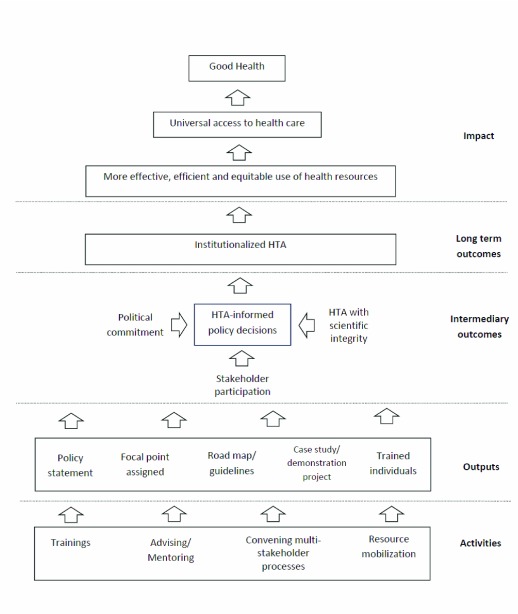
Conceptual framework for Health Technology Assessment (HTA) capacity development programs in LMICs.

HTA capacity building programs in LMICs introduced by HITAP and NICE International have the primary goal to institutionalize policy research as a means for achieving the efficient use of the limited resources in the UHC context. The two organizations developed theory of change as their framework to guide strategy and activities ( Figure 1). In this framework, equitable access to essential health care is an important long term aim of the capacity development effort. As suggested by the WHO, “… institutionalizing meant promoting structures and processes suitable to produce technology assessments that will be powerful in guiding policy and clinical practice towards the best possible health and cost outcomes” ^20^. This indicates that HTA processes, which include topic selection, evidence generation, appraisal, and appeal in a participatory and transparent manner, involve technical and political aspects of decision making, and therefore, the need for capacity development in both these dimensions.

Building technical capacity is relatively straightforward, as essential skills and knowledge for determining the impact of health technology can be developed through training of individual researchers. However, technical capacity for HTA practically relies on infrastructure and resources, such as data availability and management, expertise in related disciplines and collaborations, procedural guidelines to ensure research quality and protection from vested interests, and research grants, all of which are limited in LMICs ^12, 21^. Across the political dimension, HTA is not merely research but also a policy tool, since it generates evidence to inform policy decisions and practice. Institutionalizing HTA requires the capacity to connect the research community with the complex policymaking sphere a difficult task in resource-poor countries ^22^ and even in developed economies ^23^.

In target LMICs, the capacity building programs provide training, advising and mentoring staff to transfer expertise and knowledge on multidisciplinary research and policy development from HITAP, NICE International and their network organizations. Other activities include convening stakeholder-participatory processes of technology assessment and mobilization of financial resources, materials, expertise and information to support HTA introduction in target countries. The deliverables include HTA policy statements, road maps, government own resources committed, method and process guides, demonstration projects, case studies and trained technical officers, researchers and policy makers. In the next step of capacity development, it is expected that HTA-informed policy will be expanded and regularly introduced, as long as the three conducive elements of political commitment, stakeholder engagement and scientific integrity can be sustained.

## HITAP and NICE experiences

This paper illustrates key features of five programs that were purposively selected as case studies that capture different levels of priority setting capacity and achievement of the programs so far.

### Development of Maternal and Child Health Voucher Scheme in Myanmar

Financed by Global Alliance for Vaccine and Immunization (GAVI)’s Health System Strengthening program, a voucher scheme to improve maternal and child health (MCH) has been introduced in Myanmar since 2010. HITAP was invited by the WHO to provide technical support to the MOH to formulate financing strategy to address priority health problems ^24^. Through a consultation process, the use of vouchers as financing approach to facilitate access to MCH services, provided by skilled birth attendants, was agreed upon by decision makers and partner organizations. A series of operational research was conducted in 2010 and 2011 to inform the scheme design and implementation, such as target population, voucher distributors, benefit package, value of vouchers, payment mechanisms and communication guidelines. Besides, cost-effectiveness analysis played a crucial role in the policy adoption, since its findings suggested that the scheme would offer value for money in saving lives of mothers and newborns ^25^. Since May 2013, a pilot scheme has been introduced in one township ^26^.

The capacity development in Myanmar did not aim to institutionalize HTA, but strengthening evidence-based policymaking capacity through the engagement of decision makers and key stakeholders in every step of the operational study, as well as conventional training on health systems research, economic analysis, and public communication. It was expected that in long term such an experience would have spill-over effects on other policy issues ^25^. However, for such spill-over effects to be achieved and sustained, development partners who drive the majority of health spending in the country, would have to show political commitment to using evidence and due process in priority setting and to offer continual technical and financial support. Myanmar illustrates the important role of external partners in cultivating evidence-informed decision making in low income settings.

### Strengthening economic evaluation for EPI in the Philippines

HTA has been introduced in the Philippines since 1999 to inform coverage decisions for medicines, medical devices and procedures under PhilHealth, the national insurance scheme ^27^. In 2013, as the Department of Health (DOH) adopted economic evaluation as a tool for the development of the national formulary ^28^, it sought technical support from HITAP.

With grant from the Rockefeller Foundation, assessments of Pneumococcal conjugate virus (PCV) and Human Papillomavirus (HPV) vaccines for the national EPI were selected as demonstration studies. Aiming at transferring HITAP’s experience on vaccine assessment, Thai modelers provided guidance, access to existing economic evaluation models, and supervision to Filipino researchers. The studies suggested that both vaccines were cost effective, but countrywide HPV immunization program might be unaffordable ^29^. The DOH decided to scale up PCV vaccine coverage, while requested reanalysis of HPV vaccination provided on two-dose schedule as opposed to the three-dose schedule, as applied in the economic evaluation, since a significant decrease in budget impact was expected. 

It was considered that merely enhancing the technical aspect of decision making would be inadequate. Therefore, key players in immunization policy, including those in the DOH, national EPI and industry sector, were invited to discussion sessions on preliminary and final research findings. These aimed to fine tune the parameters and assumptions employed in the analysis, and also get policy stakeholders acquainted with HTA principles and processes. In parallel, NICE International and its partners, such as the EuroQOL team, offered training in methods of evaluation and helped review the country’s methodological and process guides for conducting HTA. Finally, with HITAP and NICE support, the Philippines joined HTAsiaLink and participated in an HTA for tobacco control initiative funded by APEC, ARCH. Capacity building through networks such as HTAsiaLink and ARCH can complement the bilateral technical cooperation activities described earlier.

Continuous support from international partners is indispensable for keeping the momentum of HTA introduction in this country. A key lesson learnt is that the development of HTA capacity can be catalyzed by beginning with a topic that the technical experts have already assessed, which is advantageous because the experts then have materials and relevant experiences to share to the local partners. This led to the completion of these projects in one year, compared to three years for the Thai studies, and two international publications in well-known journals ^29, 30^.

### Institutionalization of HTA in Vietnam

In 2012, NICE worked with Vietnam’s MoH on quality improvement in stroke management, with an emphasis on performance indicators that can improve patient care in a cost effective way. Through a multistakeholder process led by local clinicians, NICE and its NHS partners helped adapt the international evidence to the local setting and identify those simple and effective measures such as the introduction of stroke units and early patient mobilization. The quality standards derived from this process are now being rolled out in a major Hanoi public hospital with a view to further scaling up countrywide through a ministerial directive issued in 2013 ^31^.

Health economics research has existed in Vietnam since 1990s; however, a review in 2014 suggested that economic evaluations were limited in terms of scope and number ^32^. In addition, the use of cost-effectiveness evidence to inform resource allocation has not yet been formalized, owing to the lack of policy demand and link between researchers and policymakers. From 2013 HITAP and NICE International jointly convened a capacity building program for priority setting in this country, in order to address the request of the MOH, as it pursued UHC for the population. A situation analysis was conducted to gain understanding on the current mechanisms for resource allocation; the need and demand for HTA; technical capacity; and political context including key interests for connecting research with policy ^33^. The inception phase also involved raising awareness of stakeholders on HTA as a tool for priority setting. In this regard, the Vietnamese Health System and Policy Institute (HSPI) was appointed by the Health Minister as the national HTA focal point to collaborate with key stakeholders to develop HTA framework, roadmap, strategy and guidelines.

In 2016 the MOH commissioned HITAP to provide support the revision of the Vietnamese benefits package which led to the reform of benefit package of high-cost medicines and medical devices under the Vietnam Social Security Scheme (VSS) ^34^. This reform would save 3,335 billion VND (147 million US$) each year of VSS budget without minimising health outcomes by removing inappropriate use of high-cost medicines.

The capacity building model in Vietnam was designed to facilitate HTA institutionalization, by empowering local stakeholders. To do so, the main strategies in both policy and technical domains drew on HITAP’s and NICE’s experiences of conducting policy-oriented research in Thailand and other LMICs. Nevertheless, all responsible partners were well aware of the differences in health system context, including the political environment and available resources, and therefore, the need for adaptation of particular elements. Despite this, common principles of technical robustness, transparency, social accountability and policy relevance in establishing HTA institutes and of due process, such as systematic topic selection, conduction of research, dissemination of results and policy integration. These concepts were operationalized by ensuring political commitment, sense of ownership among local institutional partners, engagement of a broad range of stakeholders, and independent HTA processes.

Bringing all the different streams together, the value of this work lies in using analytical techniques such as HTA for technology evaluation and quality standard development for service improvement to inform prioritization decisions, in other words, decisions to invest pooled resources in alternative programs and technologies with a view to improving overall health in the context of UHC. Stronger capacity on HTA in Vietnam can help generate local evidence to support quality standard development that is relevant to the local context.

### Institutionalising HTA in Colombia: Human rights for one or for all?

At the request of the Minister of Health of Colombia and with seed funding from the World Bank and DFID, and, later, through a dedicated multi-year program (2008–2013) supported by the Inter-American Development Bank, NICE International and other partners, such as IECS and national universities, engaged with the Colombian authorities to help institutionalise the process for deriving a basic package, with an emphasis on technologies. In the context of strong political commitment for reform coming from the very top of the government, one of the triggers for the specific project and the initial invitation to NICE by IDB was perhaps the challenge set to the government by the country’s courts to integrate the two benefits packages (the less generous subsidised package with the more generous contributory one) and the procedural, technical and process problems posed by this decision ^35^.

Through a series of visits, exchanges and analyses, and with capacity mobilised from across Latin America, including Argentina’s Institute for Clinical effectiveness and Health Policy (IETS) ^36^ and in-country IDB expertise, NICE International, IECS contributed to an institutional and organisational blueprint, including an operational business plan, which evolved into legislation establishing IETS, the Institute for Health Technology Evaluation.

A parallel stream of technical work concentrated on comparative assessment of selection of Western (Oregon Medicaid; the Netherlands, UK, Australia) and Latin American (Chile, Costa Rica, Brazil, Uruguay) national systems, in consultation with the MOH officials. The analysis described listing as well as pricing and reimbursement processes across the selected countries, as well as actual listing and pricing decisions on the 20 pharmaceutical products (such as bevacizumab, insulin glargine and tacrolimus) most commonly requested through judicial challenge of insurer’s exceptional committees in Colombia.

The capacity building stream focused on building technical skills within Colombian universities and professional organisations in HTA and clinical guideline development, through training courses, hosting Colombian delegations of policy makers and researchers in the UK, and secondment opportunities for Colombian colleagues – the first director of IETS spent 6 months working at NICE International as part of his post graduate degree at the London School of Hygiene and Tropical Medicine (LSHTM).

The political momentum for such an effort came from the President himself, as described in the Presidential blog, which also quotes former British PM, Tony Blair identifying NICE as one of his most important achievements for the UK’s healthcare ^d^. It has been a tough process. Over two years after its formal launch, IETS is positioning itself in an ever changing system faced with threats to its financial sustainability. Coordinating cross government functions is a challenge for IETS, both in terms of responding in a timely fashion to policy priorities and in terms of linking its analyses to existing decision making processes currently led by different directorates across the major healthcare stakeholders ^e^. Perhaps the most important challenge, however, is the role of the courts, whose rulings on an individual basis undermine the very principle of UHC and invalidate any efforts by government to institutionalise priority setting. A recent ruling challenging the regulatory process ^f^ and a decision to mandate government to pay for an experimental treatment for a patient in a specialist centre in the US ^g^, are two examples of the challenge Colombia is facing.

### Reducing maternal deaths in Kerala: Bringing clinicians and policy makers together through an evidence-informed quality improvement process

HTA is very much about due process, with an emphasis on stakeholder engagement and participation. Kerala, a southern Indian State of 30 million people, has one of the lowest rates of maternal mortality in India. Nevertheless, with a maternal mortality rate of approximately 88 and every single death of a mother giving birth triggering wider social unrest and severe questioning of the authorities in the media ^37^, reducing maternal mortality was deemed to be the State’s first priority. With a committed Principal Secretary and support by DFID, Rockefeller’s Joint Learning Network ^38^ and the Wellcome Trust, NICE International worked with the State government and leading state professionals to develop and test out evidence-informed Quality Standards for tackling the top causes of maternal mortality, starting with post-partum haemorrhage (PPH) ^39^. As in the case of Vietnam, the emphasis was on turning evidence-informed guidelines into locally relevant measurable performance indicators, through a consultative and transparent process. This was followed by assessing how specific measures could be implemented locally, taking account of costs and feasibility issues. This led to training programmes targeting all labour room staff, the development and roll out of a new maternity register and the purchasing and distribution of new disposable delivery kits, including simple equipment for measuring blood loss.

Scale up of the standards across both public and private hospitals throughout the State begun in 2013 and early results suggest a reduction in the incidence of PPH and in mortality rates in the pilot hospitals ^40^, though it is still too early to draw final conclusions. In fact, even though the project introduced a new registry for measuring processes of care and outcomes, data availability, in particular baseline data needed in order to establish impact, proved to be an area in need of further strengthening.

The Kerala experience highlights the importance of involving healthcare professionals from the local constituency right from the beginning of any engagement. The success of the Kerala work relied heavily on the leadership of senior professionals from the Kerala Federation of OBGs, who had been leading for years on the only State-wide confidential enquiry into maternal deaths in India: “Why Mothers Die” ^39^. It was this enquiry that helped identify cost-effective measures targeting the main causes of maternal death. A proactive press following the story from the very beginning, also played an important role ^h^.

## Discussion

### Lessons learned from the capacity building programs in five LMICs

The five case studies illustrate the features of capacity building efforts for priority setting in different health system contexts. In the beginning, a situation analysis was conducted in each setting to determine political commitment, demand for priority setting, technical expertise in local institutes, as well as stakeholders’ attitudes and positions towards the introduction of formal priority setting mechanisms. Long-term availability of government budget to match with international grants was also explored, as local resources are necessary for sustainable evidence-based priority setting. Therefore, in some countries with notable constraints of resources, like Myanmar, the goal of capacity development was not HTA institutionalization, but rather to expose decision makers and technical officers with evidence-informed policymaking concept and practice.

Although the situation analysis could help identify potentially-successful countries and strategies for enhancing HTA capacity, the dynamicity in the political context and its effects on the practice of evidence-informed policy decisions seems to be unavoidable, such as in the case of Colombia. Furthermore, delays in the conduct of capacity building activities were observed in some study settings, owing to inadequate management ability of focal-point institutes. This indicates the need for monitoring and evaluation, as well as risk assessment and risk management approaches as integral components of program implementation.

The five study settings have reached different stages of HTA institutionalization. In most countries the capacity building activities delivered observable outputs, according to the conceptual framework, such as trained personnel and demonstration research projects ( Figure 1). However, the outputs obtained from program activities are inadequate for institutionalizing HTA ^41^. The most challenging task for HITAP and NICE International is to foster political commitment, stakeholder participation and technical integrity of HTA research as key factors of intermediary and long-term outcomes, i.e. sustainable use of evidence in policymaking and institutionalized HTA, respectively. Furthermore, although HTA institutes have been operating in some countries like Colombia, the capacity to ensure their performance and achievements has to continually be strengthened and the broader political commitment to supporting them, at the highest level, maintained. Through the implementation of WHA resolution 67.23, LMICs should benefit from programs and activities under the auspice of the WHO and other international partners along the lines described above. 

Literature on HTA capacity building in resource-poor countries suggests that political will, involvement of stakeholders, technical and financial support from international partners are crucial ^42– 44^. The capacity development programs managed by HITAP and NICE International contribute to the literature as such experiences help identify common success factors in the five study settings as follows:

1) The concept of HTA needs to be endorsed by senior decision makers including not only politicians but also health officers; this can be achieved building on the reputation of local as well as international partners. The importance of political buy-in at the Presidential level in Colombia and Ministerial level in Vietnam are cases in point.
2) The process of HTA capacity development should be demand-driven, based on local policy agenda in order to link HTA to policy decisions and also help build trust. In countries without demand for use of evidence in certain policy areas, long-term capacity development to encourage evidence-informed priority setting may not be worth the effort. In the Philippines, the government had a clear question (linked to vaccine listing) to be addressed. This demand triggered external support and also helped mobilize internal funds, leading to the country joining regional HTA networks and committing own resources to develop a designated HTA unit within DOH.
3) It is important that local institutional partner(s) are capable of doing technical work and of convening other stakeholders in-country as part of the HTA process. Based on our experience, many academic institutions in LMICs do not interact with decision makers, which limit their ability to contribute to policy and HTA capacity building. Thus, MOH or an MOH designated institution are involved as major partners. In Vietnam, HSPI, working closely with MOH as the latter’s technical arm, has been a most valuable partner and a focal point, designated by the government, through which capacity of local universities has been harvested and applied to the task of HTA analyses.
4) Ability of capacity building program managers to mobilize internal and external resources to support local partners is necessary. This is because health priority setting requires research in multiple disciplines which do not available in one single institute responsible for providing training courses and also for other facets of capacity building programs including management. Here the role of development partners in poorer countries such as Myanmar is critical. Without continuous support and coordination, evidence informed policy making can never become a reality relying on government efforts alone.

### Limitations of this paper

This paper offers a narrative of case studies over a short time period, making it hard to derive conclusions as to the success or failure of the capacity building model we are describing here. The information shared is in the form of direct experiences of the authors; as such, the information provided may not be neutral as it does not incorporate others’ viewpoints, for example, local partners and international organizations. However, these experiences have been published as scientific findings in peer-reviewed international journals.

Moreover, it is too early to determine health outcomes of HTA-based policies in the five study countries, and this is not the main focus of our paper. Based on our experiences, this is a time consuming and deeply political process, and HTA processes are implemented sometimes after several years of HTA evidence generation. Owing to such limitations, this paper does not offer recommendations on effective models for health priority setting in LMICs, but sharing experiences of the two organizations and lessons learned in different contexts. Perhaps the most important component of our work is to empower local apply the technical and political process of making evidence-informed decisions.

### Future challenges

The work on HTA capacity building within countries requires long-term effort and flexibility. As discussed earlier, it is difficult to plan well in advance the next steps, which is challenging to sustain such support in the long-term. For HITAP and NICE International, it has proven difficult to obtain funding for work that does not offer clear, measurable and certain deliverables. We are constantly faced with uncertainty and are subject to local champions and to political priorities guiding each country’s agenda.

A further challenge more specific to low-income countries (LICs), has to do with capacity at regional level and the degree to which HTA processes can be regional as opposed to country specific. The European model of HTA, EUnetHTA, involving different levels of activities such as joint work on HTA, methodological guidelines, information technology tools, legal framework, and institutional structure ^13, 14^, may be applicable to regions such as APEC or the Americas. Societies such as INAHTA, HTAi and ISPOR can have a role to play in capacity building. Emerging models such as HTAsiaLink are also valuable means of strengthening capacity.

Linked to the above to LICs, is the role of international donors in institutionalizing HTA as a technical and political tool to set priorities. The fact donors often control valuable resources for buying commodities (whereas MOH resources tend to be committed to infrastructure and salaries) and tend to have needed technical resources makes them the ideal conduit for HTA. However, their work concentrates on specific diseases (GF) and technologies (GAVI), making allocative efficiency considerations less applicable. With more countries interested in merging vertical programs with their own basic packages and with donor support declining, HTA is likely to become increasingly important.

A final challenge is the role of the private sector in the process of building institutional, data and technical capacity for HTA. Within a transparent framework where interests can be managed on all sides and participation is encouraged, HTA can serve as an ideal platform for the healthcare products as well as the private insurance industry, reducing uncertainty regarding market access and helping set standards for managing providers, respectively. Our experience has been that HTA is an engagement tool that can benefit private players, many of whom still see it as a cost-containment measure.

## Conclusion

Introducing evidence-informed priority setting in LMICs requires long-term, political commitment and support from politicians and senior health officers. Importantly, the direction for evidence generation and related capacity development should be shaped by local policy demand, which varies from setting to setting. Stakeholder and public participation in identifying HTA topics, conducting research and making policy decisions is a good practice. A key role of outsiders like the international units of NICE and HITAP is to provide general guidance on each step of HTA institutionalization that is relevant to conditions in particular countries. This also includes offering assistance for building technical, management and communication capacity of individuals and organizations that support the generation and use of HTA evidence in the respective settings. 

## Footnotes

^a^ http://programs.jointlearningnetwork.org/blog/2013/may/15/health-technology-assessment-useful-tool-countries-moving-toward-universal-health-c

^b^ http://www.lshtm.ac.uk/groups/griphealth/resources/grip_health_working_paper_3.pdf and references therein

^c^ http://www.nice.org.uk/Media/Default/News/NestaAlliance_and_NICE_paper.pdf

^d^See here: http://wsp.presidencia.gov.co/Prensa/2010/Septiembre/Paginas/20100921_04.aspx

^e^See for example: http://www.larepublica.co/economia/%C2%BFde-d%C3%B3nde-saldr%C3%A1-la-plata-para-cubrir-la-reforma-la-salud_41055 and here for a review of the broader challenges of the Colombian healthcare system: http://www.cgdev.org/blog/political-economy-uhc-colombia-version

^f^Constitutional Court: Sentencia C-313/14. http://www.corteconstitucional.gov.co/comunicados/No.%2021%20comunicado%2029%20de%20mayo%20de%202014.pdf

^g^ http://www.eltiempo.com/estilo-de-vida/salud/gobierno-apelara-medida-que-favorecio-trasplante-de-camila-abuabara/14802615

^h^See here: http://timesofindia.indiatimes.com/home/Kerala-health-department-has-decided-to-implement-the-quality-standard-for-maternal-care-in-8-maternity-hospitals-in-the-state-on-a-pilot-basis-this-year-This-will-be-followed-by-a-full-roll-out-to-all-maternity-hospitals-thereafter-said-Rajeev-Sadanandan-/articleshow/18036461.cms and here http://www.thehindu.com/todays-paper/tp-national/tp-kerala/clinical-guidelines-to-achieve-imr-reduction/article4916431.ece for early coverage.

## References

[1] WHO: The World Health Report 2013: Research for Universal Health Coverage. Geneva.2013 Reference Source

[2] INAHTA: HTA resources.2010; [cited 2010 November 23]. Reference Source

[3] Hailey D Babidge W Cameron A: HTA agencies and decision makers: An INAHTA guidance document.Stockholm: INAHTA.2010 Reference Source

[4] WHO Regional Committee for Pan America: Health technology assessment and incorporation into health systems. (Resolution CSP28/11), Washington, DC.2012 Reference Source

[5] WHO Regional Committee for Southeast Asia: Health intervention and technology assessment in support of universal health coverage. (Resolution SEA/RC66/R4), New Delhi.2013 Reference Source

[6] World Health Assembly: Health intervention and technology assessment in support of universal health coverage. (Resolution WHA 67.23), Geneva.2014 Reference Source

[7] Whyte P Hall C: The Role of Health Technology Assessment in Medicine Pricing and Reimbursement. Pharmaceutical Pricing Policies and Interventions. Geneva: WHO.2013 Reference Source

[8] Singer ME: Cost-effectiveness analysis: Developing nations left behind. *Pharmacoeconomics.* 2008;26(5):359–361. 10.2165/00019053-200826050-00001 18429653

[9] Tarn Y Hu S Kamae I: Health-Care Systems and Pharmacoeconomic Research in Asia-Pacific Region. *Value Health.* 2008;11 Suppl 1:S137–S155. 10.1111/j.1524-4733.2008.00378.x 18387058

[10] Oortwijn W Mathijssen J Banta D: The role of health technology assessment on pharmaceutical reimbursement in selected middle-income countries. *Health Policy.* 2010;95(2–3):174–184. 10.1016/j.healthpol.2009.12.008 20074829

[11] WHO: Making fair choices on the path to universal health coverage. Final report of the WHO Consultative Group on Equity and Universal Health Coverage. Geneva.2014 Reference Source

[12] Glassman A Chalkidou K Giedion U: Priority-Setting Institutions in Health: Recommendations from a Center for Global Development Working Group. *Global Heart.* 2012;7(1):13–34. 10.1016/j.gheart.2012.01.007 25691165

[13] European Commission: HTA Network adopts its Strategy for EU cooperation on Health Technology Assessments.2014; [cited 2014 29 November]. Reference Source

[14] Kristensen F Lampe K Chase DL: Practical tools and methods for health technology assessment in Europe: Structures, methodologies, and tools developed by the European network for Health Technology Assessment, EUnetHTA. *Int J Technol Assess Health Care.* 2009;25 Suppl 2:1–8. 10.1017/S0266462309990626 20030885

[15] HTAi: Health Technology Assessment International.2014; [cited 2015 9 January]. Reference Source

[16] Department of Health: The New NHS: modern, dependable.2007; [cited 2014 5 October]. Reference Source

[17] Tantivess S Teerawattananon Y Mills A: Strengthening cost-effectiveness analysis in Thailand through the establishment of the Health Intervention and Technology Assessment Program. *Pharmacoeconomics.* 2009;27(11):931–945. 10.2165/11314710-000000000-00000 19888793

[18] Teerawattananon Y Tritasavit N Suchonwanich N: The use of economic evaluation for guiding the pharmaceutical reimbursement list in Thailand. *Z Evid Fortbild Qual Gesundhwes.* 2014;108(7):397–404. 10.1016/j.zefq.2014.06.017 25444298

[19] Mohara A Youngkong S Velasco RP: Using health technology assessment for informing coverage decisions in Thailand. *J Comp Eff Res.* 2012;1(2):137–146. 10.2217/cer.12.10 24237374

[20] WHO: Institutionalization of Health Technology Assessment. Geneva.2001 Reference Source

[21] Iglesias CP Drummond MF Rovira J: Health-care decision-making processes in Latin America: problems and prospects for the use of economic evaluation. *Int J Technol Assess Health Care.* 2005;21(1):1–14. 10.1017/S0266462305050014 15736509

[22] Liverani M Hawkins B Parkhurst JO: Political and Institutional Influences on the Use of Evidence in Public Health Policy. A Systematic Review. *PLoS One.* 2013;8(10):e77404. 10.1371/journal.pone.0077404 24204823 PMC3813708

[23] Lehoux P Blume S: Technology assessment and the sociopolitics of health technologies. *J Health Polit Policy Law.* 2000;25(6):1083–1120. 10.1215/03616878-25-6-1083 11142053

[24] HITAP: Feasibility study of the Community Health Initiative for Maternal and Child Health in Myanmar. Nonthaburi, 2010.

[25] Teerawattananon Y Tantivess S Werayingyong P: Evidence-informed policy formulation: the case of the voucher scheme for maternal and child health in Myanmar. *WHO South East Asia J Public Health.* 2014;3(3):285–288. 10.4103/2224-3151.206751 28612813

[26] WHO: Mid-term review of Maternal and Child Health Voucher Scheme. Yedarshey Township, Nay Pyi Taw, Yangon: WHO Country Office, Myanmar,2014 Reference Source

[27] De Rosas-Valera M: Health technology assessment in the Philippines. *Int J Technol Assess Health Care.* 2009;25(Supp 1):231–233. 10.1017/S0266462309090680 19538815

[28] De Rosas-Valera M: Health technology assessment in the Philippines.2013; [cited 2015 9 January]. Reference Source 10.1017/S0266462309090680 19538815

[29] Guerrero AM Genuino AJ Santillan M: A cost-utility analysis of cervical cancer screening and human papillomavirus vaccination in the Philippines. *BMC Public Health.* 2015;15:730. 10.1186/s12889-015-2046-1 26223975 PMC4520072

[30] Haasis MA Ceria JA Kulpeng W: Do Pneumococcal Conjugate Vaccines Represent Good Value for Money in a Lower-Middle Income Country? A Cost-Utility Analysis in the Philippines. *PLoS One.* 2015;10(7):e0131156. 10.1371/journal.pone.0131156 26131961 PMC4488861

[31] Medical Services Administration: According to the Decision number 4858/QD-BYT December 03, 2013 of Minister of Health, Vietnam. Hanoi: Ministry of Health,2013.

[32] Tran BX Nong VM Maher RM: A systematic review of scope and quality of health economic evaluation studies in Vietnam. *PLoS One.* 2014;9(8):e103825. 10.1371/journal.pone.0103825 25122180 PMC4133226

[33] Hernandez-Villafuerte K Li R Towse A: International Decision Support Initiative (iDSI): Mapping of priority-setting in health in 17 low and middle countries across Asia, Latin America, and Africa. London: Office of Health Economics,2015 Reference Source

[34] Teerawattananon Y: Chapter 17 More Than A List: Reforming a Country’s Health Benefit Package-- A Rigorous Approach to Tackle Costly Overutilization.In *What’s In What’s Out: Designing Benefits for Universal Health Coverage.*A Glassman, U Giedion, and P Smith, Editors, Brookings: Washington D.C.,2017.

[35] Yamin A Parra-Vera O: How do courts set health policy? The case of the Colombian Constitutional Court. *PLoS Med.* 2009;6(2):e1000032. 10.1371/journal.pmed.1000032 19226184 PMC2642877

[36] NICE: NICE International Assists the Colombian Ministry of Social Protection.2009; [cited 2014 2 December]. Reference Source

[37] Rajagopal K: Where grieving mothers struggle to find answers. The Hindu,2013; [cited 2014 29 November]. Reference Source

[38] Perabathina S: Developing quality standards for post-partum haemorrhage to reduce maternal mortality in Kerala.2012; [cited 2014 29 November]. Reference Source

[39] Paily V Ambujam K Thomas B, eds.: Why Mothers Die Kerala 2006 – 2009. Second Report of Confidential Review of Maternal Deaths, Kerala 2006 to 2009. Kerala Federation of Obstetrics & Gynaecology: Kerala,2012 Reference Source

[40] Maya C: Kerala’s MMR comes down to 66.2014; [cited 2014 29 November]. Reference Source

[41] Oortwijn W Broos P Vondeling H: Mapping of health technology assessment in selected countries. *Int J Technol Assess Health Care.* 2013;29(4):424–434. 10.1017/S0266462313000469 24290336

[42] Teerawattananon Y Tantivess S Yothasamut J: Historical development of health technology assessment in Thailand. *Int J Technol Assess Health Care.* 2009;25(Supplement 1):241–252. 10.1017/S0266462309090709 19527543

[43] Kaló Z Bodrogi J Boncz I: Capacity Building for HTA Implementation in Middle-Income Countries: The Case of Hungary. *Value Health Reg Issues.* 2013;2(2):264–266. 10.1016/j.vhri.2013.06.002 29702875

[44] Doaee Sh Olyaeemanesh A Emami Sh: Development and implementation of health technology assessment: a policy study. *Iran J Public Health.* 2013;42(Supple 1):50–54. 23865016 PMC3712594

