# Supplementary material for: Health Technology Assessment capacity development in low- and middle-income countries: Experiences from the international units of HITAP and NICE
Source: F1000Res. 2017 Dec 11;6:2119. [Version 1] doi: 10.12688/f1000research.13180.1 (PMC5749126; doi:10.12688/f1000research.13180.1)
Supplement: Peer review history

# Peer review: Referee response for version 1

**DOI:** 10.5256/f1000research.14299.r28888

**Published:** 2017-12-28

**Authors:** Justice Nonvignon, Rebecca Addo

**Affiliations:** 1 Department of Health Policy, Planning and Management, University of Ghana, Accra, Ghana; 2 Centre for Health Economics Research and Evaluation, University of Technology, Sydney, NSW, Australia

**Abstract**

 “Such experiences suggest that it is not only technical capacity, for example analytical techniques for conducting economic evaluation, but also management, coordination and communication capacity that support the generation and use of HTA evidence in the respective settings"

 The above statement is not exactly clear from the country write ups. Perhaps authors could stress on each component (i.e. management, coordination and communication) in each case, by pointing out clearly how this happened for each case in the narrative. For example authors made mention of some delays in the conduct of capacity building activities in some settings due to inadequate management ability of focal point institutes. This is not clearly presented in the narrative. We suggest this be clearly outlined in the narrative for each case or for those that it is applicable before those conclusions are made in the abstract.

**Main text**

 The objectives of the paper do not come out clearly. Since the focus of the paper is on HTA capacity building, it would be great to tease out for each case:

1. What kind of trainings were given
2. The baseline skills of staff trained e.g. for researchers, did they have any basic understanding of HTA?
3. How these people were selected
4. If they required to have some particular basic skills before they qualified to be trained

 

 For each case study, readers would benefit from a highlight

1. The stage of HTA institutionalisation they were in
2. The challenges of the capacity development project
3. Supportive factors
4. The type of stakeholders that were involved in the process
5. Participation of stakeholders; their involvement and how that facilitated the process (this is done very well for the Philippines case study)

 

 For the Colombian case, readers would benefit from a brief comment on the types of national health insurance that existed, how HTA is been used for each and its challenges like the numerous context in courts, highlighting on the reasons for the context, before this is introduced under the discussion section. As it stands now, readers will need priori knowledge of the Columbian health system and national health insurance scheme to understand and appreciate the context under which the discussion is made.

**Discussion**

 The paper could benefit from the following discussion points (that is if authors have such information)

1. Since for each case study, each country was at a different stage of HTA and its use in decision making, it would be great to highlight on the skill set that was already available for countries such as the Philippines and Columbia, and the difference it made in terms of knowledge transfer
2. Did the context under which HITAP and NICE went to develop capacity in each case affect the whole process of HTA institutionalization, including capacity development: in terms of acceptance, preparations for it, etc. Were the challenges, available skills, supportive factors and stakeholder participation differ from case to case especially in terms of the stage of HTA that they were in?

Authors need to review the text for some language errors. For instance, there are some typos under limitations section on page 8 and future challenges section on page 9 and some incomplete sentences.

We have read this submission. We believe that we have an appropriate level of expertise to confirm that it is of an acceptable scientific standard.

# Peer review: Referee response for version 1

**DOI:** 10.5256/f1000research.14299.r28885

**Published:** 2017-12-21

**Authors:** Kanchan Mukherjee

**Affiliations:** 1 Centre for Health Policy, Planning and Management, School of Health Systems Studies, Tata Institute of Social Sciences (TISS), Mumbai, Maharashtra, India

This article is a descriptive account of five case studies involving the work of NICE International and HITAP in LMICs. This being an opinion piece not all arguments are supported by evidence from literature. The Theory of Change (ToC) model is excellently conceived and can be a very useful tool for future analysis of HTA work in these countries.

 However, this paper would benefit from the following:

1. There needs to be updated information in places. eg. page 4, 2 ^nd^ line, mentions country partners of NICE International and HITAP as of 2015. Considering the fact that this is end of 2017, an updated information would be better appreciated. Also, in page 5, in the section on Philippines, it would be good to update whether the HPV vaccination schedule was adapted as a two day/three day schedule. Currently, it reads incomplete.
2. The limitations of this paper are well articulated especially the acknowledgement that other view points exists. The author’s mention in the limitations that “However, these experiences have been published as scientific findings in peer-reviewed international journals”. In this context, a list of references of these experiences/other view points (perhaps one/two from each country) can be included in references.
3. There are some minor editing/typological errors which can be corrected.

With the above changes the paper can be indexed.

I have read this submission. I believe that I have an appropriate level of expertise to confirm that it is of an acceptable scientific standard.
